# Estimation of Variance Components for Growth Traits in Composite Beef Cattle Accounting for Heterosis and Recombination

**DOI:** 10.3390/genes17020173

**Published:** 2026-01-31

**Authors:** Gabriel C. Medeiros, Camila S. Mussi, Fernanda H. F. Fafarão, Elisângela C. M. Oliveira, Rafael Espigolan, Joanir P. Eler, Gabriela Giacomini, Fernando Baldi, José Bento S. Ferraz, Luis T. Gama, Hinayah R. Oliveira, Luiz F. Brito

**Affiliations:** 1 Department of Veterinary Medicine, School of Animal Sciences and Food Engineering, University of São Paulo, Pirassununga 13635-900, SP, Brazil; gabrielcostamedeiros@usp.br (G.C.M.); camilasmussi@alumni.usp.br (C.S.M.); fernandahvaladefigueiredo@gmail.com (F.H.F.F.); limattos@usp.br (E.C.M.O.); joapeler@usp.br (J.P.E.); fbaldi@usp.br (F.B.); hrojasde@purdue.edu (H.R.O.); 2 Department of Animal Sciences, Purdue University, West Lafayette, IN 47907, USA; britol@purdue.edu; 3 Department of Animal and Biological Sciences, Federal University of Santa Maria, Palmeira das Missões 98300-000, RS, Brazil; rafael.espigolan@ufsm.br; 4 International Montana Breeders Association, Mogi-Mirim 13800-000, SP, Brazil; gabriela@montana.org.br; 5 Faculty of Veterinary Medicine, University of Lisbon, 1300-477 Lisbon, Portugal; ltgama@fmv.ulisboa.pt

**Keywords:** crossbred animals, beef cattle, genomic selection

## Abstract

**Background/Objectives**: Accurate estimates of variance components are essential in breeding programs. In this context, the main objective of this study was to estimate variance components for growth traits in the Montana Composite^®^ beef population, which was developed in Brazil by crossing various taurine and indicine breeds. After 30 years of selection, the impact of recombination, heterosis, and inbreeding may have influenced the genetic background of the population. **Methods**: We analyzed data of birth weight, weaning weight, post-weaning weight gain, and yearling weight using 124,255 phenotypic records, 193,129 pedigree records, and 3911 genotyped individuals. Ten single-trait animal models (M1–M10) were compared, differing in the relationship matrix (pedigree- or genome-based relationships) and the inclusion of direct/maternal breed composition, heterosis, and recombination effects. **Results**: Models incorporating genomic information consistently yielded better fit and lower residual variances than pedigree-based models, highlighting the advantage of genomic information in capturing Mendelian sampling and realized genetic relationships. The inclusion of heterosis effects improved model fit and led to a partial reallocation of genetic variance from additive to non-additive components. In contrast, the inclusion of recombination effects in the models minimally influenced variance component estimates. Nevertheless, more complex models affected animal rankings and altered the breed composition of top-ranked selection candidates, with selection overlap between pedigree- and genomic-based evaluations ranging from moderate to high. **Conclusions**: Overall, genome-based models accounting for breed composition, heterosis, and recombination provided the most robust variance component estimates and the best support for long-term selection goals in the studied tropical composite beef cattle population.

## 1. Introduction

Accurate estimates of variance components is a fundamental element of quantitative genetics and genomic evaluations, as these components describe how the total phenotypic variance of a trait is partitioned into genetic and non-genetic sources. In animal breeding, variance components typically include additive genetic variance, which determines the response to selection, non-additive genetic variance arising mainly from dominance and epistatic interactions, and residual variance associated with environmental and unknown effects. In genomic evaluations, the estimation of these components further depends on the realized genetic relationships among individuals, which are more accurately captured using dense molecular marker information. Reliable variance component estimates are therefore essential for unbiased prediction of breeding values and for designing efficient selection strategies in livestock populations [1]. Historically, variance partitioning was estimated using analysis of variance (ANOVA), which equates expected mean squares to estimate the contribution of each factor to total phenotypic variance, guiding efficient sampling schemes [2]. While useful for balanced datasets, ANOVA’s application to unbalanced data yields highly complex estimators and no uniformly best estimators exist [3,4]. Data used in animal breeding programs are usually unbalanced due to missing observations, uneven subclass sizes, and overlapping contemporary groups. To address these limitations, Patterson and Thompson [5] developed the Restricted Maximum Likelihood (REML), a method that maximizes the likelihood of linear combinations of observations that account for fixed effects, producing unbiased variance-component estimates in unbalanced data designs [3]. The REML equations are solved iteratively, with the Expectation–Maximization (EM) algorithm offering high numerical stability even when covariance matrices are nearly singular, albeit at the cost of slow convergence [6]. On the other hand, the Average Information (AI) algorithm converges faster for routine applications but depends on heuristic criteria that can lead to slowdowns or divergence in complex models [6].

Estimating variance components in crossbred or composite populations introduces further complexity, as non-additive genetic effects, including heterosis and recombination effects, as well as genotype–environment interactions, all contribute to greater phenotypic variation, complicating the precise partitioning of the total variance and the accurate estimation of additive genetic variance. For over three decades, geneticists have investigated the impacts of crossbreeding on genetic evaluations in both beef and dairy cattle [7,8,9,10]. Studies focusing on growth traits, such as body weight at birth, weaning, and yearling; and weight gain, have consistently reported that accounting for breed effects, heterosis, and recombination in the statistical models can lead to markedly different estimates of genetic parameters [8,11,12,13]. Consequently, models that incorporate fixed regressions on breed proportion, heterosis, and recombination variables are now considered a robust framework for obtaining accurate variance component estimates in such populations [14,15].

The Montana Composite^®^ beef cattle program, initiated in 1994 in southeastern Brazil, was developed by crossing *Bos taurus indicus* (primarily Nellore) and various *B. taurus taurus* beef cattle breeds, to harness heterosis while maintaining adaptability to tropical environments [14,15]. Although variance components for various traits have been periodically updated in this population, the underlying models are still based on those proposed in 1999 [14] and re-evaluated in 2007 [16]. These models revealed moderate narrow-sense heritability estimates alongside significant heterosis and maternal effects. However, after more than thirty years of direct selection, the population has experienced changes in recombination and inbreeding coefficients [15]. As a result, the original assumption of negligible inbreeding across the population [14] is no longer valid, and recombination effects may become more pronounced [15]. These demographic and genetic shifts underscore the critical need to update variance component estimates to accurately reflect the current genetic background of the Montana Composite^®^ beef cattle population.

More recently, the Single-step Genomic Best Linear Unbiased Prediction (ssGBLUP) method has become a gold standard method in genetic evaluations. ssGBLUP simultaneously integrates data from genotyped and non-genotyped animals via the **H** matrix [17], improving the accuracy of breeding value predictions, and contributing to reduced generation intervals, thus accelerating the rate of genetic gains [17,18,19,20,21]. Since the estimation of additive genetic variance depends on the pattern of relationships among individuals [22,23], different relationship matrices (e.g., pedigree-based relationships, **P** [24] or **H** [17]) are expected to yield distinct estimates of variance components. In this context, the main objective of this study was to estimate variance components for growth traits in Montana Composite^®^ beef cattle under ten different modeling scenarios. Pedigree-based models (M1, M3, M5, M7, and M9) and genomic models based on ssGBLUP (M2, M4, M6, M8, and M10) were compared, differing in the inclusion or exclusion of genomic relationship information as well as direct and maternal heterosis and recombination effects. This framework enables a comprehensive assessment of how genomic information and non-additive genetic effects influence variance component estimates and genetic evaluation in a composite beef cattle population under artificial selection.

## 2. Materials and Methods

### 2.1. Pedigree Information, Phenotypic, and Genotypic Data

The dataset for this study consisted of Montana^®^ Composite beef cattle animals. These animals were bred and raised on 35 farms across the Brazilian states of Rio Grande do Sul, São Paulo, Minas Gerais, Goiás, Mato Grosso do Sul, Mato Grosso, Espirito Santo, Tocantins, Sergipe, and Pará, as well as in Paraguay and Uruguay. The animals were raised in extensive production systems, primarily grazing pastures dominated by *Brachiaria* and *Panicum* grasses. Some farms provided supplementation during the dry season. Calves were born mainly between September and December, coinciding with the peak availability of high-quality forage.

Population structure analyses were carried out using the CFC software (version 1.0) [25]. The pedigree file used in this study included 193,129 animals from the Montana^®^ Composite population spanning 15 generations, with 140,439 animals with more than three known generations. Of these, 29,768 animals were identified as inbred. The total number of sires and dams recorded was 3682 and 97,939, respectively. A total of 91,508 animals had no recorded progeny. The number of founders in the pedigree was 19,191, with 1016 being founder sires. The mean number (±SD) of progeny per sire was 24.88 ± 330.6, and the mean number (±SD) of progeny per dam was 1.77 ± 1.73. A more complete overview of the population dynamics over time is provided by Oliveira et al. [15].

Phenotypic records for four growth traits were made available for 124,255 animals, including information on birth weight (BW, in kg), weaning weight (WW, in kg), yearling weight (YW, in kg), and post-weaning weight gain until yearling (PWG, in kg). The phenotypic collection protocol was standardized by the Montana Composite Program^®^, as detailed by Grigolleto et al. [26], Mourão et al. [16], and Ferraz et al. [14]. Contemporary groups (CG) were defined based on animals born on the same farm, within the same year and season, of the same sex, and within the same management group. CG with fewer than five records or without phenotypic variability and phenotypic records deviating by 3.5 standard deviations from the mean were excluded from further analyses. The descriptive statistics for the analyzed traits are shown in Table 1.

Animals were genotyped using the GeneSeek^®^ Genomic Profiler (GGP) technology (Illumina Inc., San Diego, CA, USA) at various single nucleotide polymorphism (SNP) densities (30 K, 35 K, 50 K, 777 K), and imputed to approximately 52,000 SNPs using the default options from the FImpute 2.2 software [27]. The reference population used for the genotype imputations consisted of 2016 animals genotyped using a 50 K SNP panel. The genotyped animals used in the genotype imputation analyses were distributed as follows: 16 Montana Composite^®^ animals genotyped with the Illumina BovineHD BeadChip 777 K panel (777,962 SNPs reduced to 50 K by NEOGEN, Lincoln, NE, USA), 2002 Nellore animals genotyped with the HD GGP 50 K panel (54,791 SNPs), 503 Montana Composite^®^ animals genotyped with the GGP LD BeadChip 35 K panel (35,339 SNPs), and 1390 Montana Composite^®^ animals genotyped with the GGP LD BeadChip 30 K panel (30,105 SNPs). The reference genome used during the genotype imputation procedure was ARS UCD 1.2 available in GenBank under accession number GCA_002263795.2. The quality control of the genomic data was performed using the PREGSF90 program [28], part of the BLUPF90 family software (version 2.63) [29]. SNPs located on non-autosomal chromosomes, with minor allele frequency (MAF) lower than 0.01, duplicated or unknown position in the genome, or with Mendelian conflicts exceeding 1% were excluded from subsequent analyses. Additionally, SNPs and animals with call rates lower than 0.90 and 0.95, respectively, were also discarded. After quality control, the genomic database consisted of 3911 animals and 49,457 SNPs.

### 2.2. Statistical Models

The expected breed composition of each animal in the breeding program was calculated by breed, to four decimal places, based on the genetic background of their parents. Given the broad diversity of cows that initiated the program and aiming to simplify the interpretation of the results, the founder breeds were grouped into four biological types for analytical purposes: N (*B. taurus indicus* origin, including breeds such as Brahman, Gyr, Guzerat, Nellore, Sindi, Tabapuã, and Tuli), A (*B. taurus taurus* breeds adapted to tropical climates, mainly Bonsmara, Caracu, Romosinuano, and Senepol), B (*B. taurus taurus* of British origin, such as Aberdeen Angus, Devon, Hereford, and Red Angus), and C (*B. taurus taurus* of continental European origin, including Charolais, Gelbvieh, Limousin, Marchigiana, and Simmental), according to the classification originally proposed by Ferraz et al. [14] and updated in 2024 by Oliveira et al. [15].

The expected biological type composition of all animals in the dataset, computed from pedigree information, were used as linear covariates in the mixed models. These expectations were also used to compute the expected heterosis and recombination rate of each individual, following the procedures outlined by Dickerson [30] and VanRaden and Sanders [31]. The expected heterosis (Equation (1)) and recombination rate (Equation (2)) in an individual, resulting from the heterozygous combination of biological types **i** and **j**, was computed as follows:

(1) $$Het_{ij}=1-\sum_{i=1}^{n}(\alpha_{i}^{s}\times \alpha_{i}^{d})$$

(2) $$Rec_{ij}=1-\sum_{i=1}^{n}\frac{{\left(\alpha_{i}^{s}\right)}^{2}+{\left(\alpha_{i}^{d}\right)}^{2}}{2}$$

where i and j represent two breed sources, while α^s^ and α^d^ represent the corresponding breed fraction in the sire and the dam, respectively.

The variance components for each trait were estimated based on single-trait animal models and the REML method [5,32] under BLUP [1] or ssGBLUP [18] approaches, as implemented in the BLUPF90 software [29]. Initial variance estimates were obtained using the EM-REML algorithm for the first ten iterations, and the resulting values were subsequently used as starting points for the AI-REML algorithm to improve convergence efficiency. The general single-trait animal model can be expressed as:

(3) y=Xβ+Zα+Mm+Cc+ϵ

where **y** is the vector of phenotypic observations for each trait; **β** is the vector of solutions for fixed effects, as detailed in Table 2; **α** is the vector of predictions for random animal additive genetic effect; **m** is the vector of maternal additive genetic effects; **c** is the vector of maternal permanent environmental effects; and **ϵ** is the vector of random residual terms. **X**, **Z**, **M**, and **C** are the incidence matrices relating **y** to **β**, **α**, **m**, and **c**, respectively. It was assumed that: **α** ~ N(0, **K**σ^2^_α_), **m** ~ N(0, **K**σ^2^_m_), **c** ~ N(0, **I**σ^2^_c_) and **ϵ** ~ N(0, **I**σ^2^**_ϵ_**) where σ^2^_α_ is the additive genetic variance; σ^2^_m_ is the maternal additive genetic variance; σ^2^_c_ is the maternal permanent environment variance; σ^2^_ϵ_ is the residual variance; **K** is the relationship matrix, which was either the pedigree-based matrix (**P** [24]) or the combined pedigree–genomic relationship matrix (**H** [17]); and **I** is an identity matrix.

An additive genetic covariance between direct (**α**) and maternal (**m**) additive genetic effects was also considered in the model. This covariance accounts for the potential genetic correlation between the genes expressed directly in the individual and those influencing the maternal ability, which may affect the trait of interest (e.g., early growth) through the dam’s genotype [33]. The presence of this covariance is important for accurately partitioning genetic variances and covariances and for obtaining unbiased estimates of breeding values [33,34]. Accordingly, the joint distribution of **α** and **m** was assumed to follow a multivariate normal distribution:

(4) $$\left[\begin{array}{c} \alpha \\ m \end{array}\right]∼N\left(\left[\begin{array}{c} 0\\ 0 \end{array}\right],\left[\begin{array}{cc} K\sigma_{\alpha }^{2} & K\sigma_{\alpha m}\\ K\sigma_{\alpha m} & K\sigma_{m}^{2} \end{array}\right]\right),$$

where the various effects were as previously described. The complete description of the 10 different models evaluated is shown in Table 2. These models differed in the relationship matrix used and in the inclusion of breed composition, heterosis, and recombination effects. Pedigree-based models corresponded to M1, M3, M5, M7, and M9, whereas genomic models (ssGBLUP) corresponded to M2, M4, M6, M8, and M10. All models included the fixed effects of animal age (in days) at measurement (as a linear covariate), CG, and age of the dam in months (linear and quadratic covariates), as is standard practice in beef cattle genetic evaluations [35,36]. Additionally, the models accounted for the effect of embryo transfer (yes or no) and the breed of the surrogate dam to minimize potential confounding factors, as recommended by Schaeffer and Kennedy [37].

The dam’s age was included as both linear and quadratic covariates (in months), as these effects followed a quadratic trend for the traits in the population studied, as reported by Mamani et al. [38]. Biological type effects were also incorporated in all models except M1 and M2. To avoid multicollinearity, the direct additive effect for biological type N was omitted from the model, and the effects of types A, B, and C were estimated as deviations from N [39,40]. Finally, heterozygosity and recombination effects (calculated based in Equations (1) and (2), respectively) were included in the more complex models, as specified in Table 2. The variance components estimated included additive genetic variances, maternal genetic variances, genetic covariances between direct and maternal effects, permanent environmental variances, and residual variances. The genetic parameter estimates calculated included narrow-sense direct heritabilities, narrow-sense maternal heritabilities, the proportions of the total phenotypic variance explained by maternal permanent environment effects, and the genetic correlations between direct and maternal effects [33,41].

To facilitate interpretation, the evaluated models were structured to represent increasing levels of biological and genetic complexity, as described in Table 2. The models M1 and M2 assumed the population as effectively purebred, disregarding effects of breed composition, heterosis, and recombination. The models M3 and M4 incorporated direct and maternal breed composition and pooled total heterosis, without explicitly partitioning heterosis among different biological types. The models M5 and M6 extended this framework by including direct and maternal breed composition, pooled total heterosis, and pooled total recombination effects. The models M7 and M8 further increased the models complexity by accounting for direct and maternal total and specific breed composition, total direct and maternal recombination, and heterozygosity, defined as specific direct and maternal heterosis across biological types. Finally, the models M9 and M10 represent the most comprehensive parameterization, including direct and maternal breed composition as well as specific heterozygosity and recombination effects for each biological type.

### 2.3. Model Comparison

The models with identical fixed effects and differing only in the random components, due to the use of different relationship matrices, were compared using the Akaike Information Criterion (AIC; [42]) and Schwarz’s Bayesian Information Criterion (BIC; [43]) with a threshold of 10 to define that the smaller information criterion was significantly superior [42,43]. Variance component estimates were assumed to follow a normal distribution [44,45], and 95% confidence intervals (CIs) were calculated to identify significant differences among the estimates obtained from the ten models [46]. In addition to these metrics, we evaluated the proportion of animals commonly selected across models [47,48]. M7 was used as the baseline model for comparison, as it is currently the official model adopted by the Montana Composite^®^ breeding program [14]. This model includes specific heterosis coefficients but does not incorporate recombination effects or genomic information in the estimation of variance components.

Selection was based on the top 30% of animals ranked by higher estimated breeding values (EBVs), except for BW, in which lower values are preferred due to calving ease considerations in this population [26]. This threshold reflects the actual selection criteria applied in the Montana Composite^®^ Program, where no more than 30% of the calf crop may be marketed as breeding stock, in compliance with the requirements of the Special Certificate of Identification and Production (in Portuguese: “*Certificado Especial de Identificação e Produção–CEIP*”), issued by the Brazilian Ministry of Agriculture, Livestock, and Supply (MAPA, Brasilia, DF, Brazil) [15].

Lastly, the impact of model choice on biological type representation was assessed. For each trait and model, the breed composition of the top 30% selected animals was calculated, allowing comparisons of how different models may have influenced the genetic structure of the selected population.

## 3. Results

The model comparisons based on information criteria metrics are presented in Table 3, Table 4, Table 5 and Table 6. For all traits, genomic models consistently showed better (lower) AIC and BIC values than their pedigree-based counterparts, which differed only by the relationship matrix used (**P** vs. **H**). Detailed results for AIC and BIC comparisons are provided in Table 3, Table 4, Table 5 and Table 6 and Supplementary Material File S1. These tables also report estimates of direct additive genetic variance, maternal additive genetic variance, covariance between direct and maternal genetic effects, permanent environmental variance, residual variance, direct and maternal heritabilities, the proportion of maternal permanent environmental variance, and the genetic correlation between direct and maternal effects for BW, WW, PWG, and YW across the ten evaluated models.

**Figure 1:**
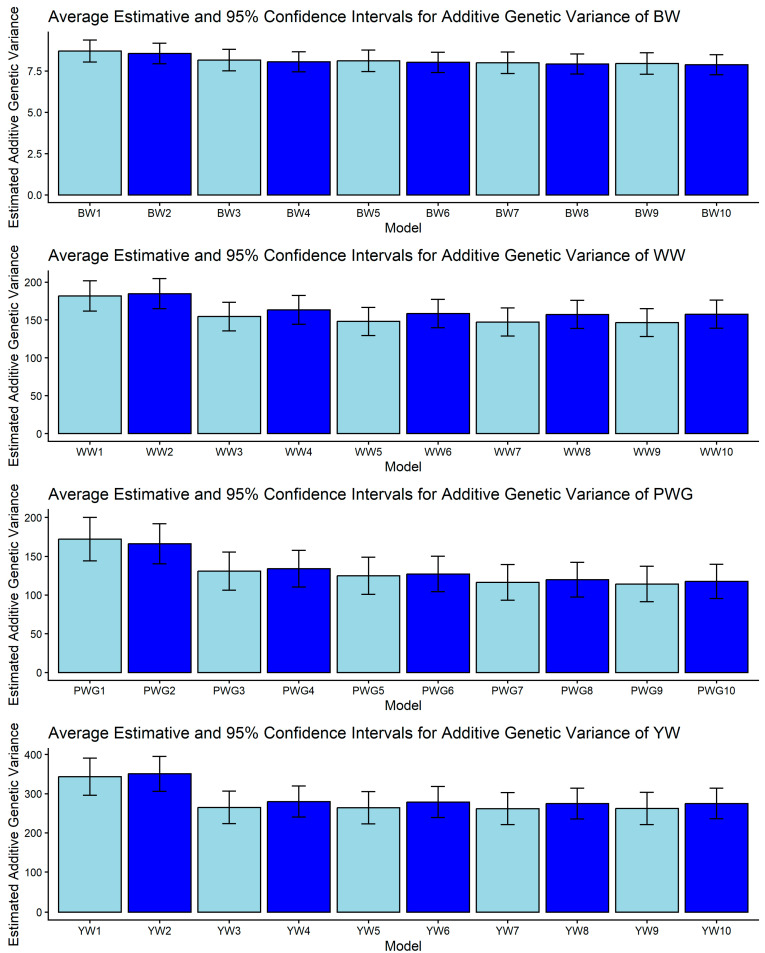
Estimates of direct additive genetic variance (means and 95% confidence intervals) obtained from models 1 to 10 (M1–M10; as described in Table 2) for four growth-related traits in Composite Montana beef cattle: birth weight (BW), weaning weight (WW), post-weaning weight gain (PWG), and yearling weight (YW).

**Figure 2:**
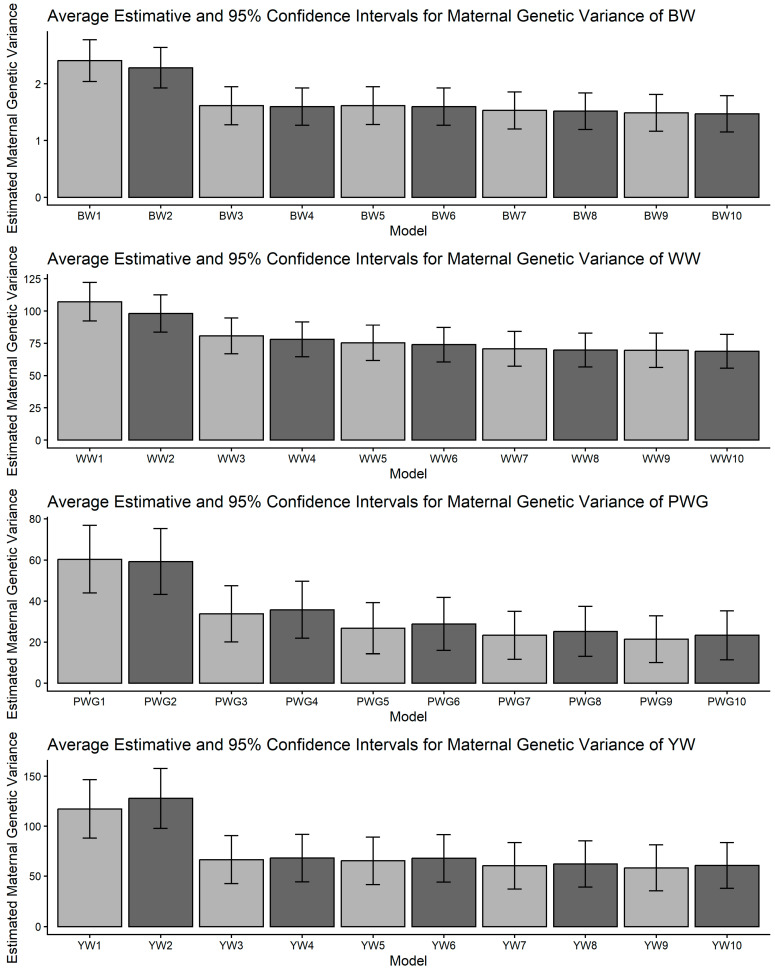
Estimates of maternal additive genetic variance (means and 95% confidence intervals) obtained from models 1 to 10 (M1–M10; as described in Table 2) for four growth-related traits in Composite Montana beef cattle: birth weight (BW), weaning weight (WW), post-weaning weight gain (PWG), and yearling weight (YW).

**Figure 3:**
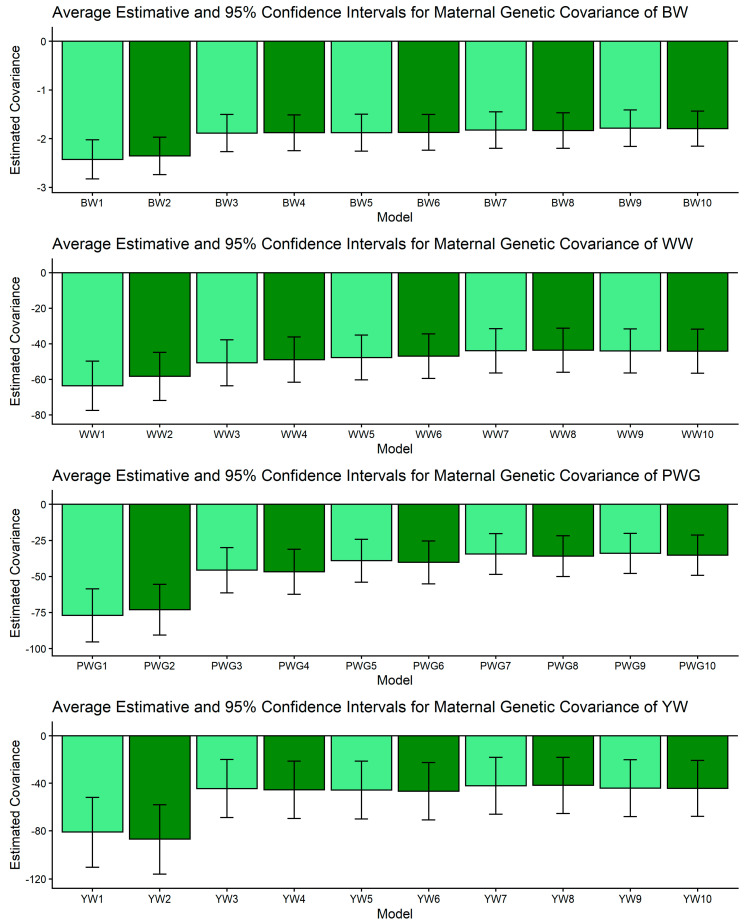
Estimates of genetic covariance between direct and maternal additive effects (means and 95% confidence intervals) obtained from models 1 to 10 (M1–M10; as described in Table 2) for four growth-related traits in Composite Montana beef cattle: birth weight (BW), weaning weight (WW), post-weaning weight gain (PWG), and yearling weight (YW).

**Figure 4:**
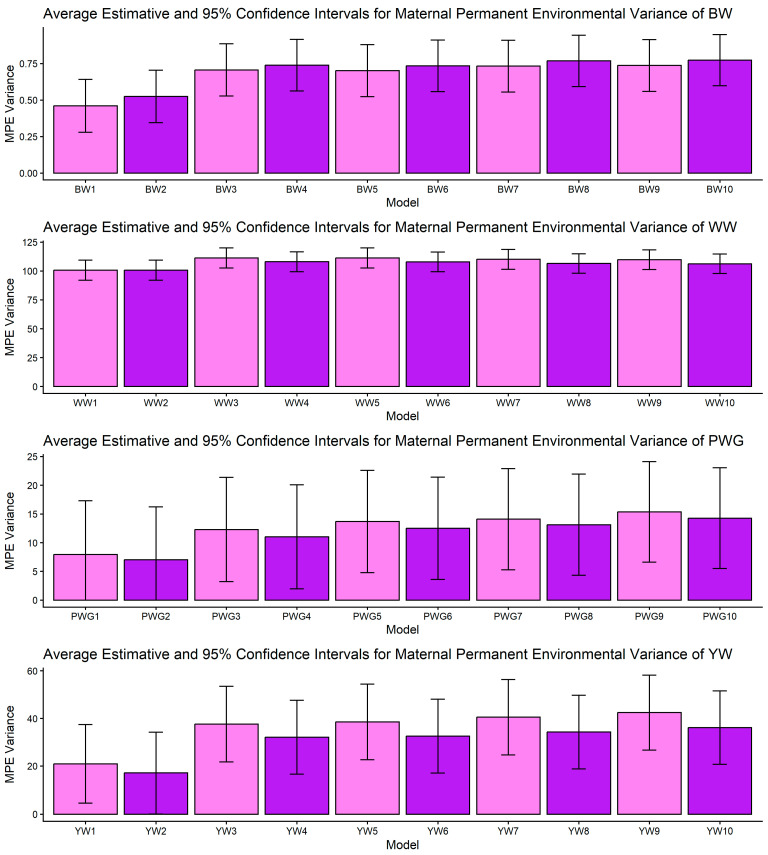
Estimates of maternal permanent environmental (MPE) variance (means and 95% confidence intervals) obtained from models 1 to 10 (M1–M10; as described in Table 2) for four growth-related traits in Composite Montana cattle: birth weight (BW), weaning weight (WW), post-weaning weight gain (PWG), and yearling weight (YW).

**Figure 5:**
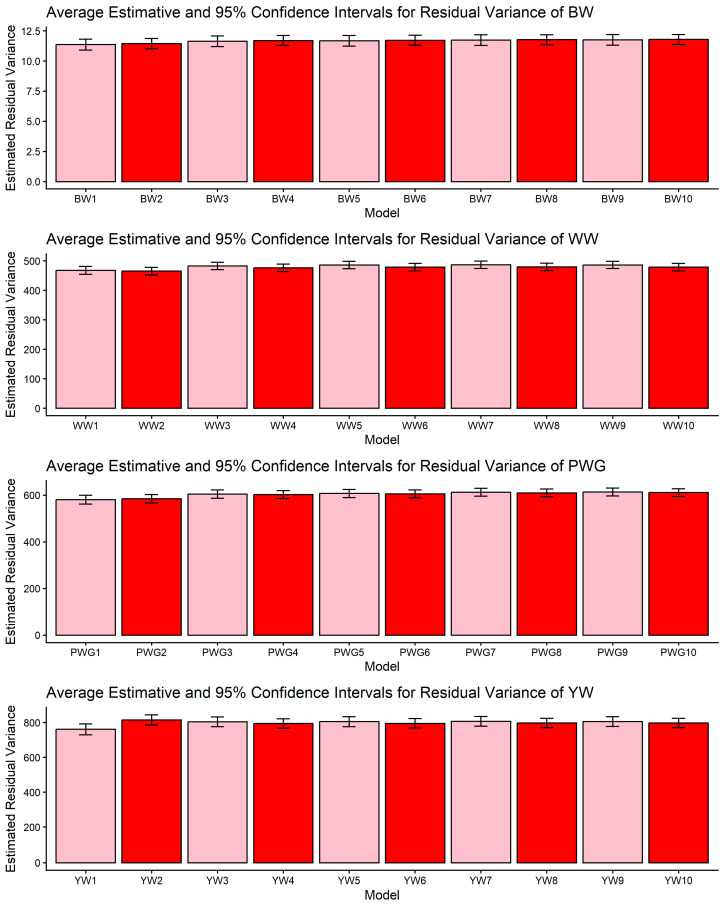
Estimates of residual variance (means and 95% confidence intervals) obtained from models 1 to 10 (M1–M10; as described in Table 2) for four growth-related traits in Composite Montana beef cattle: birth weight (BW), weaning weight (WW), post-weaning weight gain (PWG), and yearling weight (YW).

Figure 1, Figure 2, Figure 3, Figure 4 and Figure 5 display the posterior means and 95% confidence intervals of variance components across the ten models for BW, WW, PWG, and YW. Specifically, Figure 1, Figure 2, Figure 3, Figure 4 and Figure 5 present estimates of direct additive genetic variance, maternal genetic variance, covariance between direct and maternal effects, maternal permanent environmental variance, and residual variance, respectively. These figures illustrate the consistency and relative magnitude of variance components across models, as well as the effects of increasing model complexity. Estimates of maternal genetic and maternal permanent environmental variances showed considerable uncertainty, likely due to confounding arising from the low average number of progeny per dam (1.77 *±* 1.73 calves/dam), but only 163 cows had more than 10 calves.

**Figure 6:**
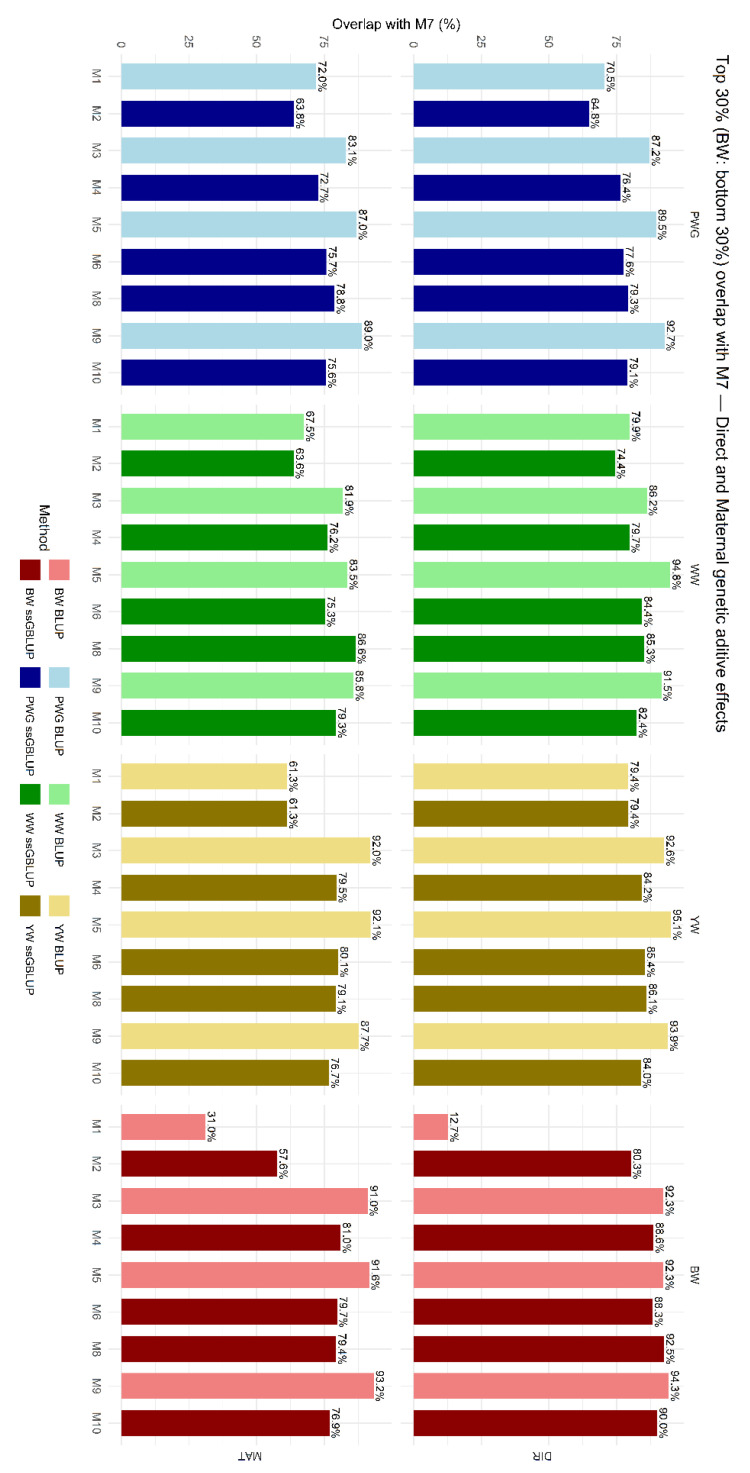
Proportion of animals commonly selected across models among the top 30% for estimated breeding values (EBVs) for direct (DIR) and maternal (MAT) additive genetic effects compared with M7. Birth weight (BW—kg), weaning weight (WW—kg), post-weaning weight gain (PWG—kg), yearling weight (YW—kg). M1-M10 are defined in Table 2.

The overlap in selected animals among models is shown in Figure 6, which presents the proportion of animals commonly selected within the top 30% based on EBVs for direct and maternal additive genetic effects. On average, genomic models (M2, M4, M6, M8, and M10) selected fewer animals in common with M7. M1 and M2, which did not account for breed composition, heterosis, and recombination, showed the greatest divergence in terms of selected CEIP candidates.

**Figure 7:**
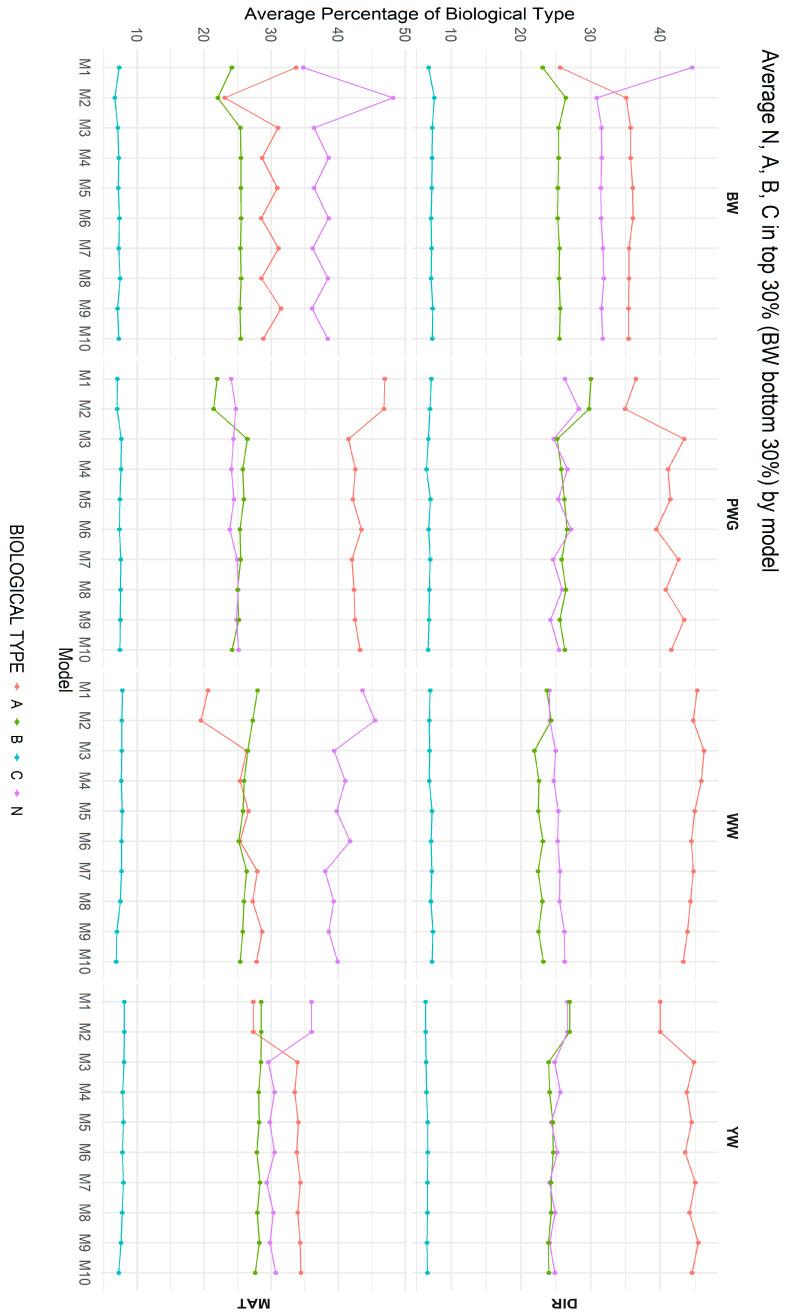
Average biological type composition of animals selected as CEIP (Special Certificate of Identification and Production) candidates under the 10 models for direct (DIR) and maternal (MAT) additive genetic effects. Birth weight (BW—kg), weaning weight (WW—kg), post-weaning weight gain (PWG—kg), yearling weight (YW—kg); direct percentage of the Zebu biological type (N); direct percentage of the adapted biological type (A); direct percentage of the British biological type (B); direct percentage of the Continental biological type (C). M1–M10 are defined in Table 2.

Figure 7 summarizes the average biological type composition of animals selected as CEIP candidates under each of the ten models, for both direct and maternal additive genetic effects. The most pronounced differences were observed between models that ignored breed composition (M1 and M2) and those that explicitly accounted for it (M3 through M10). Among Models M3 to M10, differences in breed composition were smaller but varied across traits, indicating that population structure and trait-specific genetic architecture influence selection outcomes.

## 4. Discussion

The comparison among models differing in the inclusion of genomic relationships, heterosis, and recombination effects highlights how model specification influences the partitioning of phenotypic variance in composite beef cattle populations. Rather than merely improving statistical fit [17,19,49], the incorporation of genomic information fundamentally alters how genetic covariance among individuals is represented, allowing Mendelian sampling and realized relationships to be more accurately captured [50]. This has direct implications for the estimation of additive genetic components, particularly in populations with complex breed composition, where pedigree-based expectations may not fully reflect realized genetic similarity. The observed changes in additive genetic variance across models (Table 3, Table 4, Table 5 and Table 6) are therefore best interpreted as a consequence of reweighting familial relationships under genomic information, rather than as simple increases or decreases in genetic signals [51]. In this context, the relative stability of residual variance, as shown in Figure 5, across model specifications suggests that genomic information primarily redistributes variance among biological components, instead of absorbing unexplained noise. This pattern is consistent with quantitative genetics theory and previous applications of ssGBLUP in heterogeneous beef cattle populations [17,21], reinforcing the importance of jointly modeling genomic relationships and breed composition effects when evaluating growth traits in composite breeds.

Model comparison in this study was not based on likelihood ratio tests or associated *p*-values because variance components were estimated under an REML-based framework, in which likelihoods are conditional on the fixed effects and therefore not comparable across models with different fixed effect specifications. In addition, hypothesis testing for variance components is inherently problematic because these parameters are constrained to be non-negative and often lie on the boundary of the parameter space, leading to nonstandard asymptotic distributions of test statistics and invalid classical *p*-values [52,53,54]. Furthermore, several of the evaluated models were not strictly nested, as they differed simultaneously in fixed effect structure and biological interpretation. For these reasons, inference was based on information criteria (AIC [42] and BIC [43]), confidence intervals, and a comparative evaluation of parameter estimates, including changes in predicted breeding values for commonly selected animals as well as the stability of breed composition among these animals, rather than on formal hypothesis testing, to avoid misleading conclusions regarding model superiority or parameter significance.

A limitation of this study was the relatively low proportion of genotyped animals (3.1%), which implies that the **H** matrix relied predominantly on pedigree information for most individuals. Consequently, part of the improvement observed when using genomic relationships may reflect increased connectedness and more accurate pedigree information within genotyped families, rather than the exclusive contribution of genomic data. Therefore, the advantages attributed to the genomic model should be interpreted with caution. These results represent an intermediate stage of genomic implementation in the Montana Composite^®^ population, and expanded genotyping across families and generations is expected to enhance the robustness and generalizability of genomic-based inferences in future genetic evaluations.

Beyond the relationship matrix, including heterosis and recombination effects produced modest but meaningful changes in the genetic parameter estimates. Crossbreeding exploits heterosis due to increased heterozygosity [41,55]. Models that incorporated linear heterozygosity covariates for individual and maternal heterosis captured this non-additive effect. We observed that models with heterosis (M7–M10) fit marginally better than models without it. For instance, the estimated additive genetic variance often increased, and the residual variance decreased when heterosis was not modeled (Figure 1), implying that some phenotypic variation was reattributed from the residual due the heterosis term. This agrees with the expectation that ignoring heterosis can inflate the additive genetic variance [7,30,31]. In practical terms, inclusion of heterosis means that crossbred animals are correctly penalized by the model. In our data, however, the numerical impact was relatively small. This is consistent with Akanno et al. [55], who noted that properly accounting for heterosis had “debatable effects” on EBV accuracy, and with Stock et al. [56] who reviewed that dominance-based heterosis models often resulted in only slightly higher EBV reliabilities than purely additive genetic models.

Recombination effects (the breakdown of favorable gene combinations, a form of epistatic loss) were even subtler. Following Dickerson [30] and Kinghorn [57], we included a recombination loss parameter in some models. The results showed negligible differences whether recombination was modeled or not: additive genetic and residual variances changed by less than 5%, as shown in Table 3, Table 4, Table 5 and Table 6 and Supplementary Material File S1. This suggests that recombination loss was minor in this population, as also reported by Roso et al. [58] for weight gain in crossbred cattle. In practice, this indicates that the simple additive and heterosis framework was enough for our data, and explicit recombination terms contributed little to the variance components. Kinghorn [57] predicted that recombination often has limited expression in quantitative traits, and our findings align with this theory.

### 4.1. Confidence Intervals of Estimated Variance Components

Across all four growth traits (BW, WW, PWG, and YW) the pairwise CI comparisons for residual variance did not reveal significant differences among the models (Figure 5). This result indicates that differences in model specification mainly reallocate variance among direct and maternal components rather than altering the estimated environmental noise. The stability of σ^2^_e_ is important because it suggests that observed changes in heritabilities or additive genetic variances are not artifacts of a changing error term but reflect redistribution among biological components. Similar robustness in the residual has been reported in other multi-component model comparisons in beef cattle [59]. Although the numeric reduction in the estimates of σ^2^_e_ in the more complex models (M5–M10) were not statistically significant, they may be partly explained by the heterogeneity of production environments across farms, which likely amplified the standard errors of the estimates. The Montana Composite^®^ population is raised under highly diverse environmental conditions, a factor known to influence the expression of growth phenotypes, as previously reported for this population [60,61].

Additive genetic variance showed a trait-specific pattern of sensitivity to model choice, as shown in Figure 1. For BW, no pair of models produced non-overlapping CIs, indicating that σ^2^_a_ for BW is relatively robust to the inclusion of heterosis, recombination, or genomic relationships. This robustness is plausible biologically because BW tends to have strong direct genetic control and has been repeatedly observed as less model-sensitive than maternal components in beef populations [26,62]. For WW, the only significant contrast reported among estimated direct additive genetic variances was between M2 and M9: WW2 vs. WW9 = [165.047, 204.553] vs. [128.113, 164.867]. The reduction of σ^2^_a_ from M2 to M9 corresponds to the change in relationship matrix and model complexity (M2 considered the **H** matrix while M9 is a pedigree-based model with detailed direct and maternal biological types, heterosis, and recombination structure). Genomic relationships can reallocate variance and alter σ^2^_a_ estimates in a way that depends on the fixed structure included in the model [63].

Post-weaning weight gain and YW were the traits most sensitive to model definition. PWG displayed multiple non-overlapping contrasts: higher σ^2^_a_ in M1–M2 versus markedly lower σ^2^_a_ in models M7–M10 (models including detailed heterosis/recombination parameters). Examples include PWG1 vs. PWG7: [144.044, 200.016] vs. [93.324, 139.376] and PWG1 vs. PWG9: [144.044, 200.016] vs. [91.366, 137.174]. Likewise, YW presented contrasts such as YW2 vs. YW5: [305.920, 395.380] vs. [223.000, 305.260]. The consistent trend is that simpler or pedigree-based models yielded larger σ^2^_a_ for PWG and YW, while more complex models (e.g., those including specific heterosis and recombination effects) yielded lower σ^2^_a_ estimates. This pattern is intuitive as traits measured later in life integrate direct genetic potential together with crossbreeding benefits. When non-additive effects are omitted or insufficiently modeled, environmental variance can be misallocated to σ^2^_a_, inflating direct additive variance and heritability, as presented in Figure 1 and Figure 2. Genomic relationships can help partition realized within-family variance and can reduce pedigree-induced inflation [19,63]. Our findings underscore that genomic information and explicit crossbred terms are particularly important for post-weaning and yearling traits.

The most striking model dependencies emerged for σ^2^_m_ and derived h^2^_m_. For all traits, many pairwise comparisons indicated non-overlapping CIs and systematic reductions in σ^2^_m_ when moving from simpler models to more complex models, as presented in Figure 2 and Supplemental File S1. These model-driven reductions in σ^2^_m_ were mirrored in maternal heritability results. The directional consistency suggests that more complex models reallocate variance away from the maternal additive genetic portion into explicit heterosis, recombination, breed composition, and maternal permanent environment terms (Figure 4). Classic methodological reviews and empirical studies have emphasized the difficulty of separating maternal additive genetic effects from maternal permanent environmental effects unless a sufficient number of progeny per dam are available and the tendency of simplified models to inflate maternal variance or produce spurious negative direct–maternal genetic covariances [36,64,65,66].

The σ_am_ is key for understanding selection trade-offs. For BW, WW, and YW, pairwise CI comparisons for σ_am_ indicated no non-overlapping contrasts; estimates of σ_am_ were relatively stable across models, as shown in Figure 3. For PWG, however, significant contrasts were abundant: M1 and M2 produced strongly negative covariances (e.g., PWG1: [−95.445, −58.553]) whereas more complex models (M5–M10) produced markedly less negative values (e.g., PWG7: [−48.522, −20.344]). In dataset with limited number of offsprings per dam, simpler models can produce artifactual negative direct–maternal covariances because some maternal environmental variation is allocated into the maternal genetic category, which, by covariance with direct effects, appears as antagonism [64,65]. More complex models (e.g., M5–M10) can more accurately partition non-additive and dam-level environmental variance, especially when large and well-structured datasets are available. These models increased σ^2^_mpe_, since breed composition is consistent during the whole life of the dam, biological type information can be useful to better partitioning the σ^2^_p_. The observed attenuation of negative covariance under M5–M10 therefore suggests more biologically reasonable direct × maternal relationships for PWG. This result aligns with previous reports showing that negative σ_am_ estimates are often model-dependent and reducible by improved model specification [64,65,67] and data structure. From a selection standpoint, if σ_am_ is artifactually negative, breeders may be misled into unnecessary trade-offs. The attenuation of σ_am_ in our more complex models indicates that selection for direct post-weaning growth may carry a smaller maternal penalty than simpler models suggest.

Across all traits, the σ^2^_mpe_ presented overlap of CI, and the fact that the mean number of calves per cow in the dataset is lower than 2, points to the classic identifiability problem: when cows produce few recorded progeny the ability to separate maternal genetic variance from persistent environmental effects per dam is weak, and standard errors are large [33,65]. While some models showed apparent “absorption” of maternal variance by MPE, the high standard errors did not allow statistical separation of models. This uncertainty is important. While biological interpretation suggests that some dam-level effects are persistent (e.g., breed, long-term milk capacity), our data cannot precisely quantify that fraction. Practical solutions include collecting more repeated records per dam, incorporating auxiliary dam-level indicators (e.g., milk proxies [68]), or using random regression maternal models when data structure allows [69].

An important methodological limitation of this study concerns the identifiability of maternal genetic and maternal permanent environmental effects, as shown in Figure 2 and Figure 4. The relatively low average number of progeny per dam (1.77 ± 1.73) restricts the ability to distinguish σ^2^_am_ from σ^2^_mpe_, leading to confounding between these components. As a consequence, maternal variance estimates were associated with larger uncertainty, reflected in the wide confidence intervals and substantial overlap between effects. This limitation is inherent to the data structure rather than the modeling approach and should be considered when interpreting maternal effect estimates.

### 4.2. Animals Selected in Common and Their Breed Composition

The impact of these model choices on selection was assessed via selection overlap and breed composition shifts. We compared the top 30% of animals sorted by EBV under each model and computed their overlap. Models that differed only by the presence of a heterosis term had high overlap, in general higher than 80%, indicating that adding specific heterosis did not drastically reorder ranking of potential selection candidates. In contrast, models differing in the relationship matrix (**P** vs. **H**) had lower overlap, in general of 60–80%, as shown in Figure 6. For example, for the direct additive effect of PWG, 79.3% of the top 30% ranked by a pedigree-BLUP model were also in the top 30% of its ssGBLUP counterpart. In practice, one in five selected candidates changed. This difference means genomic relationships and associated Mendelian sampling effects changed the ranking of ~10–30% of candidates. Such differences have been reported in other studies, i.e., genomic evaluations often result in re-ranking of top candidates [70].

Breed composition among the selected animals also shifted depending on the model used, as shown in Figure 7. Montana Composite^®^ cattle were originally constrained to specific biological type ranges (e.g., maximum of 37.5% of N, and 75% of B) [14,15]. Under models including heterosis and recombination, the selected top animals showed slightly higher indicine (N) fraction and lower taurine fraction compared to models without heterosis and recombination for direct additive effect for WW. These shifts are biologically meaningful: by favoring animals with more balanced or greater indicine backgrounds, models fitting heterosis and recombination help retain genetic diversity (based on percentages of biological types more balanced) and adaptation traits. If percentages of heterosis and recombination are left unchecked, a pure additive approach might gradually erode the indicine contribution, reducing heat tolerance and vigor over time. Indeed, Vanvanhossou et al. [71] found that synthetic crossing schemes that maintain heterosis also stabilize breed composition and inbreeding (the exchanged-village bull scheme). Selection plans in Montana Composite^®^ should monitor breed fractions so that long-term breeding goals (moderate frame, tropical adaptability) are not undermined by short-term gains in a single breed component.

Biologically, the observed overlap and composition patterns offer clear practical insights. The high overlap between models differing by heterosis and recombination indicates that most top-ranked animals are robust to model choice; however, the discordant ~15–30% represent precisely those individuals whose superiority depends on crossbred merit. In breeding programs, this implies that animals deemed elite under a purebred BLUP analysis may lose rank under ssGBLUP if their advantage arises primarily from heterosis rather than additive genetic value. Breeders aware of this distinction will understand that including breed composition, heterosis, and recombination effects in the evaluation tends to favor crossbred bulls, whereas purely pedigree-based models tended to prioritize purebred animals for each trait. According to model M1, an animal should ideally exhibit a higher proportion of the N biological type at birth and a higher proportion of the A type at weaning, which is not possible as breed composition is a permanent individual characteristic. In contrast, the results indicate greater stability in breed composition among selected animals from M3 to M10 for the direct additive genetic effect, and from M5 to M10 for the maternal genetic effect. Over multiple generations, these small but consistent shifts could meaningfully alter the population’s genetic structure. Our top 30% results suggest that using a ssGBLUP model may help preserve indicine alleles more effectively than relying solely on pedigree-based evaluations. Thus, model choice carries tangible consequences for long-term breed composition and inbreeding dynamics. These results indicate that differences observed in the racial composition of selected animals are largely driven by incomplete correction for breed effects rather than true biological divergence among selection candidates, as presented in Figure 7. Once breed composition is properly accounted for, the stabilization observed across traits suggests that selection decisions are primarily guided by within biological type genetic merit, reducing unintended shifts in racial structure and improving the robustness of selection in composite populations.

These interpretations tie back to the known genetic structure and goals of Montana Composite^®^ Program. Montana Composite^®^ cattle are a cross of *B. taurus taurus* and *B. taurus indicus* breeds (primarily Angus, Hereford, Devon, Simmental, Bonsmara, Senepol, and Nellore) designed for tropical environments [26,72]. As reported by Oliveira et al. [15], decades of continuous introgression of diverse germplasm have kept heterosis high and inbreeding low. Our variance component estimates are consistent with this, showing large genetic variance and moderate heritabilities for direct and maternal effects for growth. Importantly, breeding goals emphasize maintaining hybrid vigor while improving productivity. The small breed-fraction shifts under different models highlight the need for strategic management: if selection over-focuses on traits strongly expressed by one breed (e.g., WW in Angus), the indicine fraction could drift downward, ultimately reducing heterosis [30]. Vanvanhossou et al. [71] demonstrated that synthetic breeding with controlled mating can maximize gains while keeping inbreeding at low levels and benefiting from heterosis, and our findings underscore that genomic evaluation methods can assist by making heterosis, breed composition, and recombination more explicit for selection and management decisions.

## 5. Conclusions

This study evaluated the genetic background of growth traits in the Montana Composite^®^ population using phenotypic records combined with pedigree information for all animals and genomic information from a subset of individuals, allowing the comparison between pedigree-based BLUP and ssGBLUP models. Incorporating genomic relationships and explicitly modeling direct and maternal breed composition and heterosis consistently altered the partitioning of phenotypic variance and the identification of superior animals, whereas recombination effects had minor impact across traits. Models based on the **H** matrix better represented realized genetic relationships than pedigree-based models, leading to more biologically coherent estimates in the studied composite beef cattle population. Maternal genetic effects should be interpreted with caution, as the estimation of maternal variance components was subject to substantial uncertainty given the limited average number of progeny per dam in the dataset. From a breeding perspective, these results demonstrate that ssGBLUP models integrating genomic data with breed composition effects provide a more appropriate framework for selection in the Montana Composite^®^ Program, supporting long-term genetic progress while maintaining balanced indicine–taurine ancestry and benefiting from heterosis.

## Figures and Tables

**Table 1:** Descriptive statistics of growth traits in Montana Composite cattle born between 2007 and 2022.

Trait	Mean	Minimum	Maximum	SD	CV (%)	Number of Animals	NCG
**BW**	34.69	17.00	52.00	5.09	14.67	124,255	3969
**WW**	204.36	66.00	348.00	41.09	20.11	110,733	2268
**PWG**	78.84	−87.56	249.46	48.75	61.83	49,122	2200
**YW**	291.06	92.00	519.36	65.23	22.41	49,868	1324

Birth weight (BW—kg), weaning weight (WW—kg), post-weaning weight gain (PWG—kg), yearling weight (YW—kg). Number of contemporary groups (NCG).

**Table 2:** Description of the effects included in the models used to estimate variance components in Montana Composite cattle.

	Model	Fixed Effects	Random Effects	Relationship Matrix
**BW**	**M1**	CG, DA, DA^2^, ET	a, m, mpe	**P**
	**M2**	CG, DA, DA^2^, ET	a, m, mpe	**H**
	**M3**	CG, DA, DA^2^, ET, A, B, C, MA, MB, MC, TDH, TMH	a, m, mpe	**P**
	**M4**	CG, DA, DA^2^, ET, A, B, C, MA, MB, MC, TDH, TMH	a, m, mpe	**H**
	**M5**	CG, DA, DA^2^, ET, A, B, C, MA, MB, MC, TDH, TMH, TDR, TMR	a, m, mpe	**P**
	**M6**	CG, DA, DA^2^, ET, A, B, C, MA, MB, MC, THD, TDH, TDR, TMR	a, m, mpe	**H**
	**M7**	CG, DA, DA^2^, ET, A, B, C, MA, MB, MC, TDR, TMR, DH_NXA_, DH_NXB_, DH_NXC_, DH_AXB_, DH_AXC_, DH_BXC_, DH_NXA_, DH_NXB_, DH_NXC_, DH_AXB_, DH_AXC_, DH_BXC_, MH_NXA_, MH_NXB_, MH_NXC_, MH_AXB_, MH_AXC_, MH_BXC_	a, m, mpe	**P**
	**M8**	CG, DA, DA^2^, ET, A, B, C, MA, MB, MC, TDR, TMR, DH_NXA_, DH_NXB_, DH_NXC_, DH_AXB_, DH_AXC_, DH_BXC_, DH_NXA_, DH_NXB_, DH_NXC_, DH_AXB_, DH_AXC_, DH_BXC_, MH_NXA_, MH_NXB_, MH_NXC_, MH_AXB_, MH_AXC_, MH_BXC_	a, m, mpe	**H**
	**M9**	CG, DA, DA^2^, ET, A, B, C, MA, MB, MC, DH_NXA_, DH_NXB_, DH_NXC_, DH_AXB_, DH_AXC_, DH_BXC_, DH_NXA_, DH_NXB_, DH_NXC_, DH_AXB_, DH_AXC_, DH_BXC_, MH_NXA_, MH_NXB_, MH_NXC_, MH_AXB_, MH_AXC_, MH_BXC,_ DR_NXA_, DR_NXB_, DR_NXC_, DR_AXB_, DR_AXC_, DR_BXC_, DR_NXA_, DR_NXB_, DR_NXC_, DR_AXB_, DR_AXC_, DR_BXC_, MR_NXA_, MR_NXB_, MR_NXC_, MR_AXB_, MR_AXC_, MR_BXC_	a, m, mpe	**P**
	**M10**	CG, DA, DA^2^, ET, A, B, C, MA, MB, MC, DH_NXA_, DH_NXB_, DH_NXC_, DH_AXB_, DH_AXC_, DH_BXC_, DH_NXA_, DH_NXB_, DH_NXC_, DH_AXB_, DH_AXC_, DH_BXC_, MH_NXA_, MH_NXB_, MH_NXC_, MH_AXB_, MH_AXC_, MH_BXC,_ DR_NXA_, DR_NXB_, DR_NXC_, DR_AXB_, DR_AXC_, DR_BXC_, DR_NXA_, DR_NXB_, DR_NXC_, DR_AXB_, DR_AXC_, DR_BXC_, MR_NXA_, MR_NXB_, MR_NXC_, MR_AXB_, MR_AXC_, MR_BXC_	a, m, mpe	**H**
**WW, PWG,** **YW**	**M1**	CG, AA, DA, DA^2^, ET	a, m, mpe	**P**
	**M2**	CG, AA, DA, DA^2^, ET	a, m, mpe	**H**
	**M3**	CG, AA, DA, DA^2^, ET, A, B, C, MA, MB, MC, THD, TDH	a, m, mpe	**P**
	**M4**	CG, AA, DA, DA^2^, ET, A, B, C, MA, MB, MC, THD, TDH	a, m, mpe	**H**
	**M5**	CG, AA, DA, DA^2^, ET, A, B, C, MA, MB, MC, THD, TDH, TDR, TMR	a, m, mpe	**P**
	**M6**	CG, AA, DA, DA^2^, ET, A, B, C, MA, MB, MC, THD, TDH, TDR, TMR	a, m, mpe	**H**
	**M7**	CG, AA, DA, DA^2^, ET, A, B, C, MA, MB, MC, TDR, TMR, DH_NXA_, DH_NXB_, DH_NXC_, DH_AXB_, DH_AXC_, DH_BXC_, MH_NXA_, MH_NXB_, MH_NXC_, MH_AXB_, MH_AXC_, MH_BXC_	a, m, mpe	**P**
	**M8**	CG, AA, DA, DA^2^, ET, A, B, C, MA, MB, MC, TDR, TMR, DH_NXA_, DH_NXB_, DH_NXC_, DH_AXB_, DH_AXC_, DH_BXC_, MH_NXA_, MH_NXB_, MH_NXC_, MH_AXB_, MH_AXC_, MH_BXC_	a, m, mpe	**H**
	**M9**	CG, AA, DA, DA^2^, ET, A, B, C, MA, MB, MC, DH_NXA_, DH_NXB_, DH_NXC_, DH_AXB_, DH_AXC_, DH_BXC_, MH_NXA_, MH_NXB_, MH_NXC_, MH_AXB_, MH_AXC_, MH_BXC,_ DR_NXA_, DR_NXB_, DR_NXC_, DR_AXB_, DR_AXC_, DR_BXC_, MR_NXA_, MR_NXB_, MR_NXC_, MR_AXB_, MR_AXC_, MR_BXC_	a, m, mpe	**P**
	**M10**	CG, AA, DA, DA^2^, ET, A, B, C, MA, MB, MC, DH_NXA_, DH_NXB_, DH_NXC_, DH_AXB_, DH_AXC_, DH_BXC_, MH_NXA_, MH_NXB_, MH_NXC_, MH_AXB_, MH_AXC_, MH_BXC,_ DR_NXA_, DR_NXB_, DR_NXC_, DR_AXB_, DR_AXC_, DR_BXC_, MR_NXA_, MR_NXB_, MR_NXC_, MR_AXB_, MR_AXC_, MR_BXC_	a, m, mpe	**H**

Birth weight (BW—kg), weaning weight (WW—kg), post-weaning weight gain (PWG—kg), yearling weight (YW—kg); contemporary group (CG); age of the animal at the measurement (AA—days); age of the dam (DA—days); quadratic age of the dam (DA—days^2^); embryo transfer (ET—yes or no); direct percentage of the adapted biological type (A—%); direct percentage of the British biological type (B—%); direct percentage of the continental biological type (C—%); maternal percentage of the adapted biological type (A—%); maternal percentage of the British biological type (B—%); maternal percentage of the Continental biological type (C—%); total percentage of direct heterosis pooled (TDH—%); total percentage of maternal heterosis pooled (TDH—%); total percentage of direct recombination pooled (TDH—%); total percentage of maternal recombination pooled (TDH—%); percentage of specific direct heterosis between the biological types (DH_NXA_, DH_NXB_, DH_NXC_, DH_AXB_, DH_AXC_, DH_BXC_—%); percentage of specific maternal heterosis between the biological types (MH_NXA_, MH_NXB_, MH_NXC_, MH_AXB_, MH_AXC_, MH_BXC_—%); percentage of specific direct recombination between the biological types (DR_NXA_, DR_NXB_, DR_NXC_, DR_AXB_, DR_AXC_, DR_BXC_—%); percentage of specific maternal recombination between the biological types (MR_NXB_, MR_NXC_, MR_AXB_, MR_AXC_, MR_BXC_—%); direct additive genetic effect (a); maternal genetic effect (m); maternal permanent environment effect (mpe); pedigree-based relationship matrix (**P**); combined pedigree–genomic relationship matrix (**H**). The effects of direct percentage of groups A, B, and C are computed as deviation from N (*B. taurus indicus* biological type).

**Table 3:** Estimates (±SE) of variance and covariance components for birth weight (BW) in Montana Composite cattle using different single-trait models.

Model	σ^2^_a_	σ^2^_m_	σ^2^_am_	σ^2^_mpe_	σ^2^_e_	h_d_^2^	h_m_^2^	c^2^	rg_am_	BIC	AIC
**M1**	8.71 (0.34)	2.41 (0.19)	−2.42 (0.20)	0.46 (0.09)	11.36 (0.23)	0.38 (0.01)	0.10 (0.01)	0.02 (0.01)	−0.53 (0.02)	361,721.65	361,813.8
**M2**	8.56 (0.32)	2.28 (0.18)	−2.35 (0.20)	0.52 (0.09)	11.45 (0.21)	0.38 (0.01)	0.10 (0.01)	0.02 (0.01)	−0.53 (0.02)	361,032.64	361,124.8
**M3**	8.16 (0.33)	1.61 (0.17)	−1.89 (0.19)	0.71 (0.09)	11.65 (0.23)	0.37 (0.01)	0.07 (0.01)	0.03 (0.01)	−0.52 (0.03)	360,800.68	360,986.6
**M4**	8.05 (0.31)	1.60 (0.17)	−1.88 (0.19)	0.74 (0.09)	11.70 (0.21)	0.36 (0.01)	0.07 (0.01)	0.03 (0.01)	−0.52 (0.03)	360,148.85	360,334.8
**M5**	8.12 (0.33)	1.62 (0.17)	−1.88 (0.19)	0.70 (0.09)	11.67 (0.23)	0.37 (0.01)	0.07 (0.01)	0.03 (0.01)	−0.52 (0.03)	360,762.62	360,972
**M6**	8.02 (0.31)	1.60 (0.17)	−1.87 (0.19)	0.74 (0.09)	11.72 (0.21)	0.36 (0.01)	0.07 (0.01)	0.03 (0.01)	−0.52 (0.03)	360,111.09	360,320.5
**M7**	8.00 (0.33)	1.53 (0.17)	−1.82 (0.19)	0.73 (0.09)	11.73 (0.22)	0.36 (0.01)	0.07 (0.01)	0.03 (0.01)	−0.52 (0.03)	360,396.82	360,747.0
**M8**	7.92 (0.31)	1.52 (0.16)	−1.83 (0.19)	0.77 (0.09)	11.77 (0.21)	0.36 (0.01)	0.07 (0.01)	0.04 (0.01)	−0.53 (0.03)	357,196.76	357,546.9
**M9**	7.96 (0.33)	1.49 (0.17)	−1.78 (0.19)	0.74 (0.09)	11.75 (0.22)	0.36 (0.01)	0.07 (0.01)	0.03 (0.01)	−0.52 (0.03)	360,219.37	360,663.4
**M10**	7.88 (0.31)	1.47 (0.16)	−1.79 (0.18)	0.77 (0.09)	11.79 (0.21)	0.36 (0.01)	0.07 (0.01)	0.04 (0.01)	−0.53 (0.03)	359,580.39	360,024.4

σ^2^_a_: direct additive genetic variance; σ^2^_m_: maternal additive genetic variance; σ^2^_am_: genetic covariance between direct and maternal genetic effects; σ^2^_mpe_: maternal permanent environmental variance; σ^2^_e_: residual variance; h_d_^2^: direct heritability; h_m_^2^: maternal heritability; c^2^: coefficient of maternal permanent environmental variance; rg_am_: genetic correlation between direct and maternal; BIC: Schwarz’s Bayesian Information Criterion; AIC: Akaike Information Criterion. M1–M10 are defined in Table 2.

**Table 4:** Estimates (±SE) of variance and covariance components for weaning weight (WW) in Montana Composite cattle using different single-trait models.

Model	σ^2^_a_	σ^2^_m_	σ^2^_am_	σ^2^_mpe_	σ^2^_e_	h_d_^2^	h_m_^2^	c^2^	rg_am_	BIC	AIC
**M1**	181.73 (10.26)	107.21 (7.59)	−63.57 (7.08)	100.76 (4.47)	468.08 (6.76)	0.21 (0.01)	0.12 (0.01)	0.12 (0.01)	−0.45 (0.03)	703,567.68	703,670.6
**M2**	184.80 (10.08)	98.10 (7.35)	−58.33 (6.91)	100.75 (4.41)	465.49 (6.62)	0.22 (0.01)	0.12 (0.01)	0.12 (0.01)	−0.43 (0.03)	702,655.29	702,758.2
**M3**	154.52 (9.67)	80.73 (7.08)	−50.72 (6.58)	111.28 (4.43)	482.66 (6.48)	0.19 (0.01)	0.10 (0.01)	0.13 (0.01)	−0.45 (0.04)	702,489.83	702,685.7
**M4**	163.37 (9.66)	78.02 (6.90)	−48.90 (6.51)	108.02 (4.35)	476.37 (6.42)	0.20 (0.01)	0.09 (0.01)	0.13 (0.01)	−0.43 (0.04)	701,638.34	701,834.2
**M5**	148.06 (9.43)	75.33 (6.94)	−47.70 (6.42)	111.31 (4.41)	486.30 (6.36)	0.18 (0.01)	0.09 (0.01)	0.14 (0.01)	−0.45 (0.04)	702,107.17	702,326.2
**M6**	158.45 (9.51)	73.94 (6.81)	−46.98 (6.40)	107.85 (4.34)	479.15 (6.36)	0.19 (0.01)	0.09 (0.01)	0.13 (0.01)	−0.43 (0.04)	703,146.07	703,365.1
**M7**	147.36 (9.41)	70.73 (6.84)	−43.94 (6.34)	110.10 (4.38)	486.66 (6.35)	0.18 (0.01)	0.09 (0.01)	0.14 (0.01)	−0.43 (0.04)	701,700.19	702,035.4
**M8**	157.33 (9.48)	69.78 (6.72)	−43.56 (6.32)	106.51 (4.32)	479.66 (6.34)	0.19 (0.01)	0.09 (0.01)	0.13 (0.01)	−0.41 (0.04)	700,838.95	701,174.2
**M9**	146.49 (9.38)	69.56 (6.80)	−44.02 (6.31)	109.77 (4.36)	486.40 (6.33)	0.18 (0.01)	0.09 (0.01)	0.14 (0.01)	−0.43 (0.04)	701,245.81	701,697.2
**M10**	157.63 (9.48)	68.71 (6.68)	−44.17 (6.31)	106.28 (4.29)	478.75 (6.34)	0.19 (0.01)	0.08 (0.01)	0.13 (0.01)	−0.42 (0.04)	700,393.78	700,845.2

σ^2^_a_: direct additive genetic variance; σ^2^_m_: maternal additive genetic variance; σ^2^_am_: genetic covariance between direct and maternal effects; σ^2^_mpe_: maternal permanent environmental variance; σ^2^_e_: residual variance; h_d_^2^: direct heritability; h_m_^2^: maternal heritability; c^2^: coefficient of maternal permanent environmental variance; rg_am_: genetic correlation between direct and maternal; BIC: Schwarz’s Bayesian Information Criterion; AIC: Akaike Information Criterion. M1–M10 are defined in Table 2.

**Table 5:** Estimates (±SE) of variance and covariance components for post-weaning gain (PWG) in Montana Composite cattle using different single-trait models.

Model	σ^2^_a_	σ^2^_m_	σ^2^_am_	σ^2^_mpe_	σ^2^_e_	h_d_^2^	h_m_^2^	c^2^	rg_am_	BIC	AIC
**M1**	172.03 (14.28)	60.38 (8.39)	−77.00 (9.41)	7.95 (4.77)	581.27 (9.76)	0.21 (0.02)	0.07 (0.01)	0.01 (0.01)	−0.76 (0.04)	293,058.82	293,155.2
**M2**	166.19 (13.15)	59.22 (8.17)	−73.06 (8.96)	7.02 (4.71)	584.67 (9.14)	0.20 (0.01)	0.07 (0.01)	0.01 (0.01)	−0.74 (0.04)	291,988.4	292,084.8
**M3**	131.03 (12.57)	33.78 (6.97)	−45.66 (8.04)	12.30 (4.63)	604.64 (9.06)	0.17 (0.01)	0.04 (0.01)	0.02 (0.01)	−0.69 (0.06)	292,779.07	292,961.9
**M4**	134.13 (12.06)	35.77 (7.10)	−46.73 (7.99)	11.04 (4.62)	602.79 (8.74)	0.17 (0.01)	0.05 (0.01)	0.01 (0.01)	−0.68 (0.06)	291,747.82	291,930.7
**M5**	124.83 (12.22)	26.80 (6.38)	−39.09 (7.59)	13.70 (4.55)	607.74 (8.91)	0.16 (0.01)	0.03 (0.01)	0.02 (0.01)	−0.68 (0.07)	292,704.17	292,908.6
**M6**	127.27 (11.73)	28.88 (6.57)	−40.19 (7.58)	12.51 (4.54)	606.26 (8.61)	0.16 (0.01)	0.04 (0.01)	0.02 (0.01)	−0.67 (0.08)	291,649.53	291,854.0
**M7**	116.35 (11.75)	23.32 (5.96)	−34.43 (7.19)	14.10 (4.50)	612.55 (8.71)	0.15 (0.01)	0.03 (0.01)	0.02 (0.01)	−0.66 (0.09)	292,498.13	292,810.6
**M8**	119.93 (11.38)	25.24 (6.19)	−35.89 (7.24)	13.13 (4.50)	610.29 (8.47)	0.16 (0.01)	0.03 (0.01)	0.02 (0.01)	−0.65 (0.07)	291,440.10	291,752.6
**M9**	114.27 (11.69)	21.40 (5.82)	−33.98 (7.10)	15.38 (4.46)	613.73 (8.69)	0.15 (0.01)	0.03 (0.01)	0.02 (0.01)	−0.69 (0.08)	292,292.20	292,712.7
**M10**	117.62 (11.31)	23.32 (6.08)	−35.23 (7.16)	14.27 (4.47)	611.61 (8.45)	0.15 (0.01)	0.03 (0.01)	0.02 (0.01)	−0.68 (0.07)	291,237.11	291,657.6

σ^2^_a_: direct additive genetic variance; σ^2^_m_: maternal additive genetic variance; σ^2^_am_: genetic covariance between direct and maternal effects; σ^2^_mpe_: maternal permanent environmental variance; σ^2^_e_: residual variance; h_d_^2^: direct heritability; h_m_^2^: maternal heritability; c^2^: coefficient of maternal permanent environmental variance; rg_am_: genetic correlation between direct and maternal; BIC: Schwarz’s Bayesian Information Criterion; AIC: Akaike Information Criterion. M1–M10 are defined in Table 2.

**Table 6:** Estimates (±SE) of variance and covariance components for yearling weight (YW) in Montana Composite cattle using different single-trait models.

Model	σ^2^_a_	σ^2^_m_	σ^2^_am_	σ^2^_mpe_	σ^2^_e_	h_d_^2^	h_m_^2^	c^2^	rg_am_	BIC	AIC
**M1**	343.27 (24.16)	117.29 (14.93)	−81.12 (14.91)	20.94 (8.43)	760.81 (15.72)	0.28 (0.02)	0.09 (0.01)	0.02 (0.01)	−0.40 (0.05)	318,025.78	318,122.3
**M2**	350.65 (22.82)	127.87 (15.31)	−87.07 (14.79)	17.16 (8.72)	815.11 (14.99)	0.27 (0.01)	0.10 (0.01)	0.01 (0.01)	−0.41 (0.05)	317,088.47	317,185.0
**M3**	265.08 (21.04)	66.46 (12.25)	−44.48 (12.47)	37.62 (8.12)	804.31 (14.35)	0.23 (0.02)	0.06 (0.01)	0.04 (0.01)	−0.33 (0.07)	317,338.99	317,522.1
**M4**	279.93 (20.17)	68.07 (12.15)	−45.48 (12.34)	32.09 (7.93)	794.52 (13.72)	0.24 (0.01)	0.06 (0.01)	0.03 (0.01)	−0.33 (0.07)	316,103.71	316,286.8
**M5**	264.13 (20.98)	65.31 (12.14)	−45.67 (12.42)	38.53 (8.10)	804.80 (14.32)	0.22 (0.02)	0.06 (0.01)	0.03 (0.01)	−0.34 (0.07)	317,263.36	317,468.1
**M6**	278.73 (20.13)	67.83 (12.13)	−46.81 (12.32)	32.56 (7.93)	795.22 (13.71)	0.24 (0.01)	0.06 (0.01)	0.03 (0.01)	−0.34 (0.07)	316,038.09	316,242.8
**M7**	261.99 (20.90)	60.31 (11.84)	−42.09 (12.23)	40.54 (8.08)	806.15 (14.29)	0.22 (0.02)	0.05 (0.01)	0.03 (0.01)	−0.33 (0.07)	317,113.90	317,426.8
**M8**	274.73 (19.97)	62.13 (11.80)	−41.78 (12.09)	34.29 (7.90)	797.51 (13.65)	0.24 (0.02)	0.05 (0.01)	0.03 (0.01)	−0.32 (0.07)	315,878.46	316,191.3
**M9**	262.36 (20.91)	58.26 (11.77)	−44.09 (12.21)	42.45 (8.05)	805.63 (14.29)	0.22 (0.02)	0.05 (0.01)	0.04 (0.01)	−0.35 (0.07)	316,875.85	317,296.9
**M10**	275.01 (19.98)	60.72 (11.72)	−44.33 (12.06)	36.14 (7.87)	797.09 (13.64)	0.23 (0.01)	0.05 (0.01)	0.03 (0.01)	−0.34 (0.07)	315,645.37	316,066.4

σ^2^_a_: direct additive genetic variance; σ^2^_m_: maternal additive genetic variance; σ^2^_am_: genetic covariance between direct and maternal effects; σ^2^_mpe_: maternal permanent environmental variance; σ^2^_e_: residual variance; h_d_^2^: direct heritability; h_m_^2^: maternal heritability; c^2^: coefficient of maternal permanent environmental variance; rg_am_: genetic correlation between direct and maternal; BIC: Schwarz’s Bayesian Information Criterion; AIC: Akaike Information Criterion. M1–M10 are defined in Table 2.

## Data Availability

The data supporting the results of this article are included within the article/Supplementary Materials. The raw data cannot be made publicly available, as it is property of the Montana Composite beef cattle breeders and this information is commercially sensitive. Reasonable requests for access to the raw datasets for research purposes can be e-mailed to jbferraz@usp.br (J.B.S.F).

## Supplementary Materials

The following supporting information can be downloaded at https://www.mdpi.com/article/10.3390/genes17020173/s1. Supplementary Material File S1: Variance components estimates from models M1-M10, for birth weight (BW), weaning weight (WW), post-weaning gain (PWG) and yearling weight (YW) from Montana Composite cattle.

## Abbreviations

The following abbreviations are used in this manuscript:

BW	Birth Weight (kg)
WW	Weaning Weight (kg)
PWG	Post-Weaning Weight Gain (kg)
YW	Yearling Weight (kg)
NCG	Number of Contemporary Groups
−2logL	Negative Twice the Log-Likelihood
AIC	Akaike Information Criterion
BIC	Schwarz’s Bayesian Information Criterion
BLUP	Best Linear Unbiased Prediction
SsGBLUP	Single-Step Genomic BLUP
REML	Restricted Maximum Likelihood
EM-REML	Expectation Maximization Restricted Maximum Likelihood
AI-REML	Average Information Restricted Maximum Likelihood
SNP	Single Nucleotide Polymorphism
MAF	Minor Allele Frequency
EBV	Estimated Breeding Value
CEIP	Certificado Especial de Identificação e Produção (Special Certificate Of Identification And Production)
CG	Contemporary Group
AA	Age of the Animal at Measurement (Days)
DA	Age of the Dam (Days)
DA^2^	Quadratic Age of the Dam (Days^2^)
ET	Embryo Transfer (Yes/No)
A	Direct Percentage of Adapted Biological Type
B	Direct Percentage of British Biological Type
C	Direct Percentage of Continental Biological Type
MA	Maternal Percentage of Adapted Biological Type
MB	Maternal Percentage of British Biological Type
MC	Maternal Percentage of Continental Biological Type
TDH	Total Direct Heterosis Percentage
TMH	Total Maternal Heterosis Percentage
TDR	Total Direct Recombination Percentage
TMR	Total Maternal Recombination Percentage
DHNXA	Specific Direct Heterosis between N and A Biological Types
DHNXB	Specific Direct Heterosis between N and B Biological Types
DHNXC	Specific Direct Heterosis between N and C Biological Types
DHAXB	Specific Direct Heterosis between A and B Biological Types
DHAXC	Specific Direct Heterosis between A and C Biological Types
DHBXC	Specific Direct Heterosis between B and C Biological Types
MHNXA	Specific Maternal Heterosis between N and A Biological Types
MHNXB	Specific Maternal Heterosis between N and B Biological Types
MHNXC	Specific Maternal Heterosis between N and C Biological Types
MHAXB	Specific Maternal Heterosis between A and B Biological Types
MHAXC	Specific Maternal Heterosis between A and C Biological Types
MHBXC	Specific Maternal Heterosis between B and C Biological Types
DRNXA	Specific Direct Recombination between N and A Biological Types
DRNXB	Specific Direct Recombination between N and B Biological Types
DRNXC	Specific Direct Recombination between N and C Biological Types
DRAXB	Specific Direct Recombination between A and B Biological Types
DRAXC	Specific Direct Recombination between A and C Biological Types
DRBXC	Specific Direct Recombination between B and C Biological Types
MRNXA	Specific Maternal Recombination between N and A Biological Types
MRNXB	Specific Maternal Recombination between N and B Biological Types
MRNXC	Specific Maternal Recombination between N and C Biological Types
MRAXB	Specific Maternal Recombination between A and B Biological Types
MRAXC	Specific Maternal Recombination between A and C Biological Types
MRBXC	Specific Maternal Recombination between B and C Biological Types
a	Direct Additive Genetic Effect
m	Maternal Genetic Effect
mpe	Maternal Permanent Environmental Effect
**P**	Pedigree-Based Relationship Matrix
**H**	Combined Pedigree–Genomic Relationship Matrix
